# Dispersed origins and concentrated professional entry in Colombian men's international football, 1962–2026

**DOI:** 10.3389/fspor.2026.1946961

**Published:** 2026-09-16

**Authors:** Samuel José Gaviria-Alzate, Angie Julieth Gómez-Restrepo, Wilder Geovanny Valencia-Sánchez

**Affiliations:** 1 Faculty of Education and Social Science, Tecnológico de Antioquia, Institución Universitaria, Medellín, Colombia; 2 Instituto Universitario de Educación Física y Deporte, Universidad de Antioquia, Medellín, Colombia

**Keywords:** association football, athlete migration, Colombia, geographical concentration, professional entry, sport geography, talent pathways

## Abstract

Colombia combines marked territorial diversity with a professional football system concentrated in a limited set of urban centres, making it an informative case for separating where elite players originate from where they enter professional competition. This retrospective observational study examined the geographical origins and professional entry points of Colombia's senior men's internationals and treated temporal composition and region-position patterning as secondary analyses. We assembled 306 unique players selected across 27 FIFA World Cup and Copa América final tournaments between 1962 and 2026, triangulated public documentary records and applied variable-specific two-source verification. Among 161 players with verified paired data, 72 (44.7%) made their professional debut in their department of birth. In the directly comparable domestic-debut subset of 154 players, birthplaces spanned 20 departments whereas professional debuts spanned 14; the three most frequent departments accounted for 58.4% of birthplaces and 70.1% of debuts. Regional shares of newly selected players fluctuated rather than shifting progressively: the Pacífica share peaked in 1990–2009 and then declined, while Caribe returned to approximately its initial share. This conclusion was unchanged when 2014 replaced 2010 as the second temporal cut-point. In the secondary region-by-position analysis, the association was small, *χ*²(6, *N* = 287) = 17.85, *p* = 0.007, Cramér's V = 0.18; after Bonferroni correction only the excess of Caribe-born forwards remained. The findings establish a reproducible territorial baseline: elite origins were more dispersed than professional entry, temporal composition was non-monotonic and regional origin provided little basis for positional profiling. Longitudinal data on upbringing, youth clubs, academies and migration are required to identify the mechanisms underlying professional-entry concentration.

## 1 Introduction

Colombia provides an informative case for examining how a geographically dispersed population is connected to a concentrated professional football system. Senior internationals have emerged from municipalities across the Andean interior and the Caribbean and Pacific coasts, yet professional clubs and entry opportunities have historically clustered in a smaller set of metropolitan centres. Studying both birthplace and professional debut therefore permits a direct test of whether the territorial reach of elite origins is broader than the geography through which players first enter paid competition.

This case also requires care in the use of regional categories. Colombia's natural regions differ in settlement patterns, urbanisation, economic conditions, ethnic composition, transport connectivity and access to football organisations, but each region is internally heterogeneous and administrative boundaries do not delimit homogeneous cultures. In this study, Andina, Caribe and Pacífica are aggregate geographical units used to describe distributions and retain adequate analytical denominators. They are not treated as biological, ethnic or culturally uniform populations.

Football converts unevenly distributed local participation into a highly concentrated professional labour market. The places in which elite players are born, the places where they acquire sporting resources and the organisations that authorise entry into paid competition are not necessarily the same. Treating these locations as interchangeable obscures the institutional work through which mobility, recruitment and selection organise sporting opportunity. Research on football labour has shown that player markets operate through spatially differentiated circuits and that mobility is a routine, consequential feature of professional careers (1, 2). Yet much research on the geography of talent still uses birthplace as if it were the developmental environment itself.

The birthplace-effect literature offers an important but limited starting point. Elite athletes are not distributed proportionally across settlement sizes or population densities, although the direction and magnitude of these associations vary by sport, sex and national system (3–5). A systematic review in football found no universal community-size advantage; participation, local sociodemographic composition, proximity to development centres and access to facilities interacted in context-specific ways (6). Birthplace is therefore best treated as a geographical marker whose social meaning requires explanation, not as an exposure that produces talent.

Studies that separate birthplace from the site of development reinforce this caution. In the Netherlands, associations between municipal talent yield and urbanity weakened after football participation, household income and population composition were considered (7). In a Spanish academy, municipalities of development tended to be larger, denser and closer to the club than municipalities of birth, while geographical transitions were more common among later academy entrants (8). These findings suggest a relational geography of opportunity: local environments may support participation, but access to professional institutions can require movement towards organisational centres.

Professional debut is not equivalent to development, but it is an observable milestone of entry into professional competition. A player may learn the game in one territory, be recruited through another and first appear professionally in a third. The distinction matters sociologically because spatial concentration of professional entry can coexist with dispersed origins. It may reflect club location, scouting reach, transport, family relocation, academy capacity or unequal selection thresholds. Evidence from an Australian talent-search programme, for example, indicates that athletes outside the metropolitan region faced higher empirical selection thresholds despite nominally common criteria (9). The present study cannot test such mechanisms, but it can establish whether origins and professional entry are territorially aligned.

Evidence from the Global South strengthens this labour-market interpretation. In Brazilian elite men's futsal, Souza et al. (10) showed that clubs and employment opportunities were concentrated in the South and Southeast and that domestic mobility reproduced unequal regional career possibilities. Colombia presents a complementary question in an outcome-selected national-team sample: whether internationals originated across a broader territorial base than the departments in which their first professional appearances were recorded. We refer to this observable pattern as the spatial concentration of professional entry. It represents a difference between two pathway markers and does not identify the actors or mechanisms that produced it.

Geography also intersects with social classifications of players. Research on positional “stacking” examines the systematic allocation or representation of socially perceived groups in particular playing roles. From early North American studies to football analyses in England and Germany, positional concentrations have been interpreted through institutional expectations and racialised stereotypes rather than innate group aptitude (11–13). Colombia offers a related test of regional profiling: regional origin is sometimes invoked in football discourse when describing playing style and positional suitability. Because this proposition has not been tested systematically, the present study treats it as a hypothesis rather than as an established characteristic of players from particular regions.

Within Colombian football discourse, regions are also associated with stylised player profiles, for example, Caribbean attacking creativity, Pacific speed or physicality, and Andean tactical organisation and national-team history is sometimes narrated as a progressive movement away from the Andean interior towards Pacific and Caribbean origins. These claims are socially produced descriptions, not assumptions of innate regional aptitude. They are testable only as patterns of representation: the first through a region-by-position contingency analysis and the second through the composition of newly selected players over time.

Taken together, these strands support a relational opportunity-structure framework. Football opportunity is not located in birthplace alone; it is organised through relations among local participation environments, family and mobility resources, scouting networks, academies, clubs and league structures. Professional debut is therefore treated as an institutionally authorised threshold into paid competition, while regional position patterns are treated as possible products of social classification and allocation rather than evidence of inherent aptitude. This framework integrates the labour-mobility, geographical-development and stacking literatures already reviewed.

Professional football pathways are embedded within a broader sport-management environment in which clubs, leagues, governing bodies and other stakeholders coordinate resources, communication and organisational priorities across increasingly globalised settings (14). Within this institutional environment, the framework distinguishes three claims that should not be collapsed. Spatial concentration is an observed distribution; unequal opportunity requires evidence that the probability or cost of access differs across territories; and social reproduction requires evidence that institutional practices sustain those differences over time. The present outcome-selected sample can establish the first and identify where the latter two should be tested, but it cannot demonstrate territorial discrimination or causal reproduction. This distinction is socially important because dispersed origins do not, by themselves, establish that professional football offers an equally accessible route to occupational or upward mobility.

Against this framework, the overarching objective was to determine how geographical origin, professional entry and secondary regional patterning intersected in the historical composition of Colombia's senior men's international team between 1962 and 2026. Specifically, we aimed to: (i) describe the distribution of players' birthplaces across municipalities, departments and natural regions; (ii) determine the correspondence between department of birth and department of professional debut and compare the territorial concentration of these markers within the same players; (iii) describe changes in the regional composition of newly selected internationals across broad analytical periods; and (iv), as a secondary exploratory analysis, test whether region of birth was associated with index playing position. We hypothesised that professional entry would be more territorially concentrated than players' geographical origins. The temporal and positional analyses were exploratory; neither was designed to infer regional aptitude, homogeneous playing cultures or the mechanisms producing the observed distributions.

## 2 Materials and methods

### 2.1 Design

This observational, retrospective and descriptive-analytical study was based on the secondary analysis of publicly available historical and biographical records. The study followed established principles for the transparent reporting of observational research and for the systematic appraisal and use of documentary sources (15, 16). No intervention or direct interaction with individuals occurred, and all information concerned public figures in their professional sporting roles. No private, confidential or sensitive personal data were collected. Because the study relied exclusively on publicly available documentary sources and involved no participant recruitment, intervention, biological material or access to private records, formal ethics review was not sought, in accordance with the applicable institutional requirements.

The design and analytical strategy were matched to these objectives. Documentary frequencies, maps and descriptive rates characterised geographical origins; the paired same-player comparison evaluated birthplace and professional-entry concentration without changing the observations being compared; unique-player period proportions described historical composition; and Pearson's chi-squared test, adjusted standardised residuals and Cramér's V evaluated the secondary region–position association. These methods reconstruct long-term distributions and associations from documentary records but do not estimate causal effects, territorial selection probabilities or individual social mobility.

### 2.2 Sample and scope

The sampling frame comprised two types of senior men's international final tournament in which Colombia participated: the FIFA World Cup final tournaments of 1962, 1990, 1994, 1998, 2014, 2018 and 2026, and the Copa América/South American Championship final tournaments of 1963 and every edition from 1975 to 2024. This yielded twenty-seven tournaments in total. The study population consisted of footballers identified in the strongest available final-squad or tournament-specific records. For 1963, because a complete official registered squad list could not be located, membership was reconstructed from tournament and match records and treated as a lower-certainty source than official final-squad documentation.

The study period began in 1962 on the basis of a predefined data-quality criterion. The 1962 FIFA World Cup was the earliest included tournament for which the squad could be verified consistently against an official tournament record and supported by sufficiently traceable biographical information. Earlier South American Championship participations were excluded because squad composition and player-level biographical data could not be verified to the same standard across sources. Their inclusion would therefore have introduced differential ascertainment and reduced comparability across the study period. The 1967 South American Championship was excluded because Colombia did not qualify for the final tournament. Qualifying competitions were also outside the sampling frame because the study was restricted to final-tournament squads.

The primary unit of analysis was the unique player, indexed to his chronologically earliest eligible tournament after sorting records by tournament year and row identifier. Players appearing in more than one tournament were therefore counted once in player-level analyses. When a player named in a preliminary squad was replaced before the tournament, the officially registered replacement was included and the withdrawn player was excluded. For example, Farid Díaz was coded in place of Frank Fabra in 2018, and Carlos Carbonero in place of Aldo Ramírez in 2014. Squad validation was consequently based on the effective final squad with confirmed pre-tournament replacements incorporated. Data collection was closed in June 2026.

### 2.3 Data sources and verification

Data were extracted and triangulated from the archives of the Colombian Football Federation; the Rec.Sport.Soccer Statistics Foundation (RSSSF), whose match line-ups were particularly important for the earlier period; official FIFA tournament materials; biographical databases; and Colombian historical newspapers, including El Tiempo, El Espectador, El Colombiano, La Patria and regional titles, particularly for disputed cases. For each player-level variable, birthplace, professional debut club and playing position, information was cross-checked across available independent sources (15). Cases in which at least two independent sources agreed were classified as meeting the two-source verification standard. Where discrepancies occurred, greater weight was given to primary, official or contemporaneous sources, and all adjudications were documented in a decision log.

An evidence-grade variable was retained to distinguish cases supported by official records, contemporaneous press reports, biographical chronicles or first-person accounts from cases relying principally on secondary biographical databases. The inferential sensitivity analysis was restricted to players whose birthplace met the two-source verification standard.

Squad composition was validated using the strongest available tournament-specific sources. The 2026 squad was checked against the official final-squad announcement of the Colombian Football Federation, while the other tournaments were checked against official, contemporaneous or specialist historical records according to availability. For 1963, membership was reconstructed from tournament and match records rather than a complete official registered list. Across the full database of 306 unique players, birthplace met the two-source verification standard for 264 players (86 per cent). Analyses involving broader valid regional classifications reported their exact denominators and were accompanied by a two-source-verified sensitivity analysis. Data collection, source verification and database coding were completed in June 2026, which served as the study data cut-off date.

### 2.4 Variables and coding

Birthplace was recorded at municipality and department level and grouped into Colombia's widely used natural-region classification: Andina, Caribe, Pacífica, Orinoquía, Amazonía and Insular. Region was derived deterministically from department, and the complete coding crosswalk is supplied in Supplementary Data S1. The regional categories were used as aggregate spatial groupings rather than proxies for homogeneous culture, ethnicity or biology; the analysis recognises substantial internal heterogeneity and boundary cases within each region. Birthplace, place of upbringing and location of professional debut were treated as conceptually distinct constructs because they need not coincide; however, information on upbringing was not sufficiently complete or standardised for inclusion. Ethnicity, Afro-Colombian identity and socially assigned skin-colour categories were not coded. The study does not treat human populations as biologically discrete races; such terminology is retained only when it forms part of the original title of a cited publication.

The professional debut club was defined as the club for which the player made his first official competitive appearance in a professional division; friendly matches were excluded. When the first professional appearance occurred outside Colombia, it was retained as a separate “abroad” category rather than assigned to a Colombian department. Department of professional debut was treated as an indicator of entry into professional competition, not as a complete measure of the player's developmental environment.

Playing position was taken from the official squad list for the index tournament and coded into four categories: goalkeeper, defender, midfielder and forward. For the seven players classified in different positions across later tournaments, the position recorded at the index tournament was retained. Because this four-category scheme does not reliably distinguish between attacking and defensive midfielders, the regional proposition was tested as an association between region of birth and the four official position groups rather than as a forced offensive/defensive dichotomy.

Departmental population was obtained from the 1985 Colombian census (17) for the descriptive calculation of internationals per million inhabitants. The source values used in the calculations are reproduced in Supplementary Data S1, S2. Use of a common census denominator facilitates comparison across departments but does not account for each player's birth cohort. The resulting rates are ecological descriptive indicators, not incidence rates, estimates of individual probability or causal measures of regional talent production.

### 2.5 Statistical analysis

Absolute and relative frequencies were calculated for categorical variables. The association between region of birth and playing position was examined using Pearson's chi-squared test of independence, after confirming that expected cell frequencies were adequate for asymptotic inference (18). Cell-level departures from independence were subsequently evaluated using adjusted standardised residuals, which approximate standard normal deviates under the null hypothesis (19). Because twelve cells were inspected, family-wise type I error was controlled using a Bonferroni correction; the resulting two-sided threshold was *p* < 0.0042, equivalent to |z| > 2.86 (20). Cramér's V was reported as the measure of association strength (18).

Territorial mobility was described using players for whom both department of birth and department of professional debut met the two-source verification standard (*n* = 161). To compare territorial concentration using identical observations, a secondary analysis was restricted to players with two-source-verified birth and professional-debut departments whose debut occurred in Colombia (*n* = 154). For birthplace and professional debut within this directly comparable sample, we calculated the number of represented departments and the proportion accounted for by the three most frequent departments. Departmental birthplace distributions and population-normalised rates used all two-source-verified birthplace records (*n* = 264; 263 Colombian-born players contributed to departmental rates), whereas the professional-debut distribution used all two-source-verified debut records (*n* = 174). The region-by-position analysis was restricted to players with a valid Andina, Caribe or Pacífica region of birth and a coded position at the index tournament (*n* = 287).

For the exploratory temporal analysis, each player was counted once at his chronologically earliest eligible tournament. Players with a valid Andina, Pacífica or Caribe classification were grouped into 1962–1989, 1990–2009 and 2010–2026. The first cut coincides with Colombia's return to the FIFA World Cup in 1990 after a 28-year absence. The second was a pragmatic decade boundary chosen to separate the pre-2010 tournaments from the contemporary cycle while retaining adequate denominators (*n* = 95, 109 and 83); because no eligible tournament occurred in 2010, the last group effectively begins with Copa América 2011. These broad bins were intended to reduce tournament-level volatility and provide interpretable comparisons, not to define fixed developmental eras. To assess dependence on the second boundary, we repeated the summary with Colombia's 2014 World Cup return as the alternative cut-point, yielding 1962–1989, 1990–2013 and 2014–2026. Both analyses were descriptive; no formal test of temporal trend was performed.

The overall significance level for the secondary region-by-position association was set at *α* = 0.05. Analyses were conducted in Python 3 using pandas, NumPy and SciPy; figures were produced using Matplotlib. Given the observational and ecological design, the analyses identify associations and descriptive geographical patterns but do not support causal inference regarding regional aptitude, recruitment decisions or developmental quality.

## 3 Results

### 3.1 The sample

The database comprised 306 unique players across twenty-seven tournaments. Birthplace met the two-source verification standard for 264 players (86%), playing position was available for 299 players, and 287 players had a valid Andina, Pacífica or Caribe classification. All 287 also had a coded index position and were included in the secondary exploratory region-by-position analysis. These same 287 region-classified players formed the basis of the secondary descriptive temporal analysis. The denominator varied across other analyses according to the availability and verification status of the required variables.

### 3.2 Geographically dispersed origins and concentrated professional entry

Among 161 players with two-source-verified paired data, 72 (44.7%) made their professional debut in their department of birth, 82 debuted in another Colombian department and seven debuted abroad. In the directly comparable subset of 154 players whose professional debut occurred in Colombia, birthplaces spanned 20 departments whereas professional debuts spanned 14. The three most frequent birth departments accounted for 90 of 154 players (58.4%), compared with 108 of 154 (70.1%) for the three most frequent debut departments. Professional entry was therefore more territorially concentrated than geographical origin within the same set of players (Figure 1). Chocó recorded the highest descriptive rate of internationals per million inhabitants using the common 1985 census denominator (47.8 per million), compared with 3.4 per million in Bogotá D.C. (Table 1, Figure 2).

**Figure 1:**
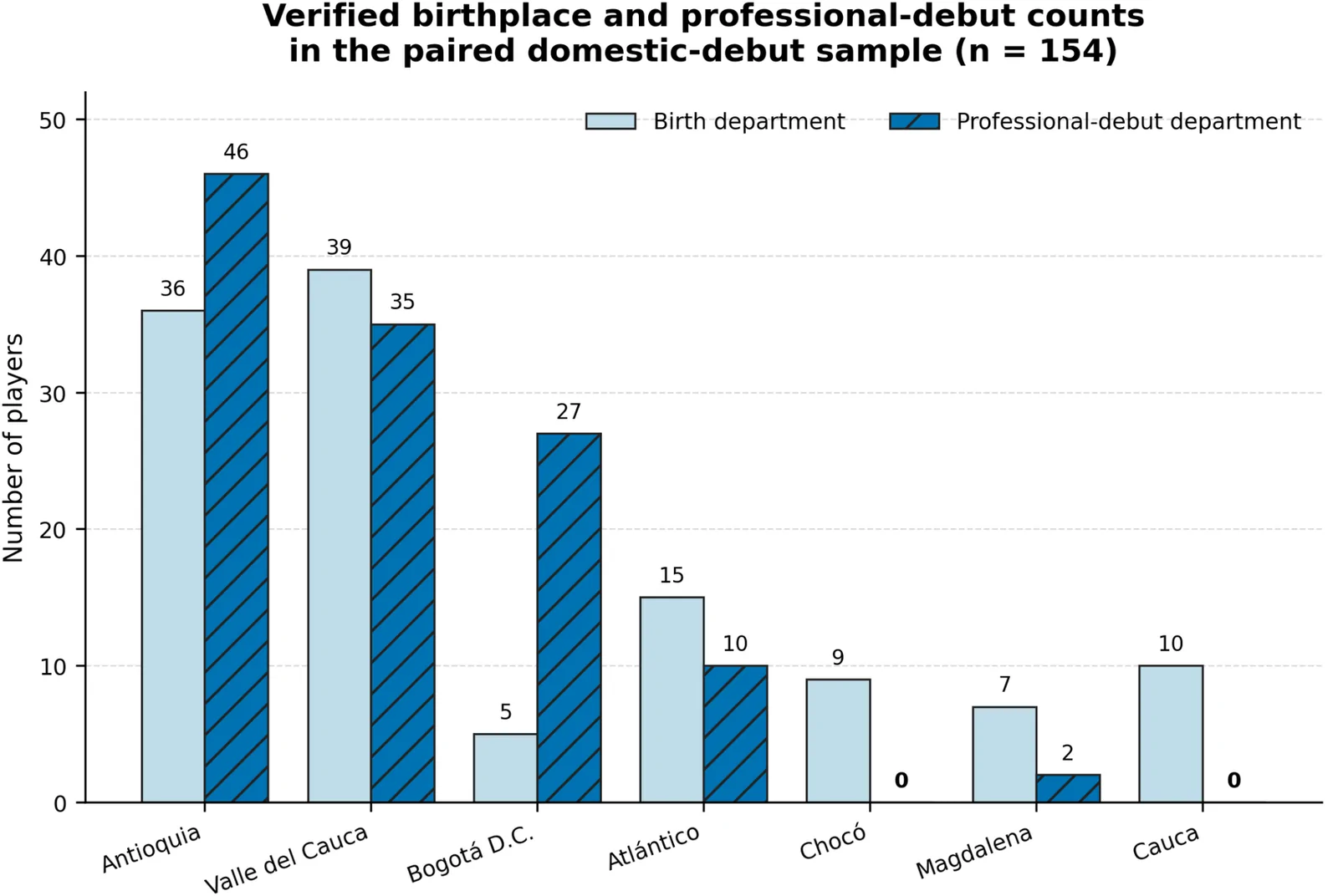
Two-source-verified birthplace and professional-debut counts for seven departments in the directly comparable domestic-debut sample (*n* = 154). All players had verified birth and debut departments and made their professional debut in Colombia; both bar series therefore refer to the same players. Across the full paired sample (*n* = 161), seven additional players debuted abroad. In the domestic-debut sample, birthplaces spanned 20 departments and professional debuts 14; the three most frequent departments accounted for 58.4% of birthplaces and 70.1% of debuts.

**Table 1:** Number of internationals per million inhabitants in the ten departments with the highest descriptive rates, with Bogotá D.C. included as the comparison used in the text.

Department	Internationals (*n*)	Population (1985)	Rate per million
Chocó	15	313,567	47.8
Valle del Cauca	65	3,027,247	21.5
Magdalena	14	890,934	15.7
Antioquia	63	4,067,664	15.5
Cauca	13	857,751	15.2
Atlántico	22	1,478,213	14.9
Nariño	12	1,085,173	11.1
La Guajira	3	299,995	10.0
Quindío	3	392,208	7.6
Caldas	6	883,024	6.8
Bogotá D.C.	14	4,123,262	3.4

The 1985 census is a common denominator and is not matched to players' birth cohorts.

**Figure 2:**
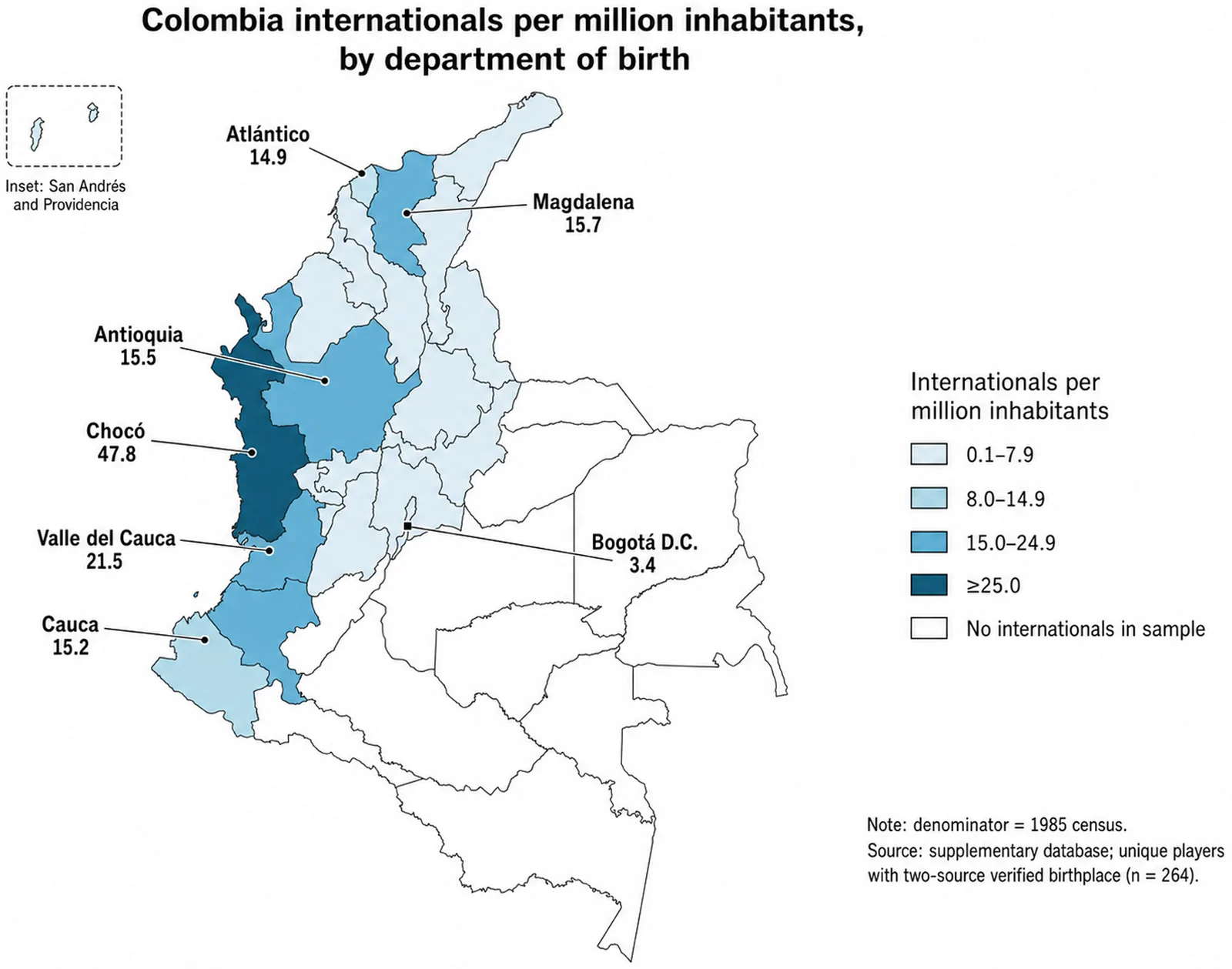
Colombia internationals per million inhabitants by department of birth, using the 1985 census denominator. Departments are grouped into four solid-colour rate categories (0.1–7.9, 8.0–14.9, 15.0–24.9 and ≥25.0), with a separate category for departments with no internationals in the sample. Birthplace met the two-source verification standard for 264 players; 263 Colombian-born players contributed to departmental rates. The seven departments discussed in the text are labelled with their corresponding rates.

### 3.3 Regional composition over time

The regional composition of players appearing in the study sample for the first time varied across the three analytical periods (Figure 3). In 1962–1989, Andina accounted for 40 of 95 players (42.1%), Pacífica for 32 of 95 (33.7%) and Caribe for 23 of 95 (24.2%). In 1990–2009, Pacífica recorded its highest proportion, with 54 of 109 players (49.5%), followed by Andina with 42 of 109 (38.5%) and Caribe with 13 of 109 (11.9%). In 2010–2026, Andina accounted for 36 of 83 players (43.4%), Pacífica for 27 of 83 (32.5%) and Caribe for 20 of 83 (24.1%). The regional proportions therefore fluctuated rather than following a monotonic transition. The sensitivity analysis using 2014 as the second cut-point produced the same interpretation: Andina accounted for 42.1%, 40.5% and 40.9% across 1962–1989, 1990–2013 and 2014–2026; Pacífica for 33.7%, 47.6% and 31.8%; and Caribe for 24.2%, 11.9% and 27.3%, respectively (Supplementary Data S3).

**Figure 3:**
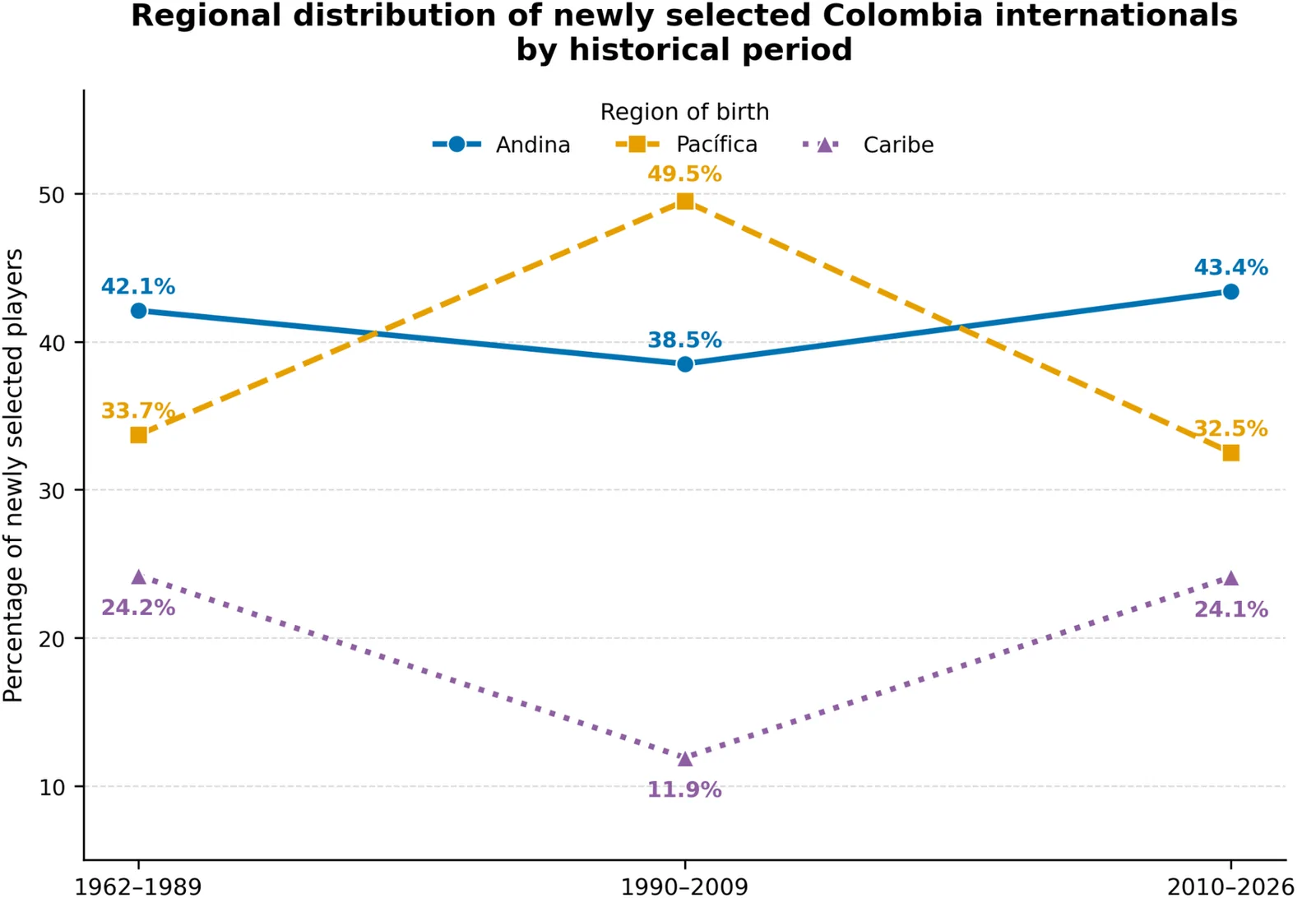
Regional distribution of newly selected Colombia internationals by historical period. Each player was indexed to his chronologically earliest eligible tournament. Values show the percentage of unique players first selected in each period whose birthplace was classified in the Andina, Pacífica or Caribe region (*n* = 287). The periods were 1962–1989 (*n* = 95), 1990–2009 (*n* = 109) and 2010–2026 (*n* = 83). The figure describes changes in regional composition and should not be interpreted as a population-adjusted production rate or a smooth annual trend.

### 3.4 Region of birth and playing position

In the secondary positional analysis, region of birth was associated with playing position, *χ*²(6, *N* = 287) = 17.85, *p* = 0.007, with a small effect size (Cramér's V = 0.18; Table 2). Adjusted standardised residuals identified several nominal cell-level departures from independence. After Bonferroni correction for twelve inspected cells, however, only the Caribe–forward cell exceeded the corrected threshold (*z* = 3.77; Figure 4). Forwards accounted for 46% of Caribe-born players, compared with 18% of Andina-born players. The pattern remained in the sensitivity analysis restricted to two-source-verified birthplaces (*n* = 263), *χ*²(6) = 15.45, *p* = 0.017, Cramér's V = 0.17; the Caribe–forward cell again exceeded the corrected threshold (*z* = 3.51).

**Table 2:** Region of birth by playing position among unique players with a valid Andina, Caribe or Pacífica classification and coded index position (*n* = 287).

Region	Goalkeepers	Defenders	Midfielders	Forwards	Total
Andina	11 (0.13)	43 (2.12)	43 (0.47)	21 (−2.79)	118
Caribe	4 (−0.56)	9 (−2.47)	17 (−0.79)	26 (+3.77)	56
Pacífica	11 (0.32)	33 (−0.12)	40 (0.16)	29 (−0.25)	113
Total	26	85	100	76	287

Adjusted standardised residuals are shown in parentheses. With Bonferroni correction for twelve cell comparisons, |z| > 2.86 indicates statistical significance. *χ*²(6) = 17.85, *p* = 0.007, Cramér's V = 0.18. The two-source-verified sensitivity analysis (*n* = 263) yielded *χ*²(6) = 15.45, *p* = 0.017 and Cramér's V = 0.17.

**Figure 4:**
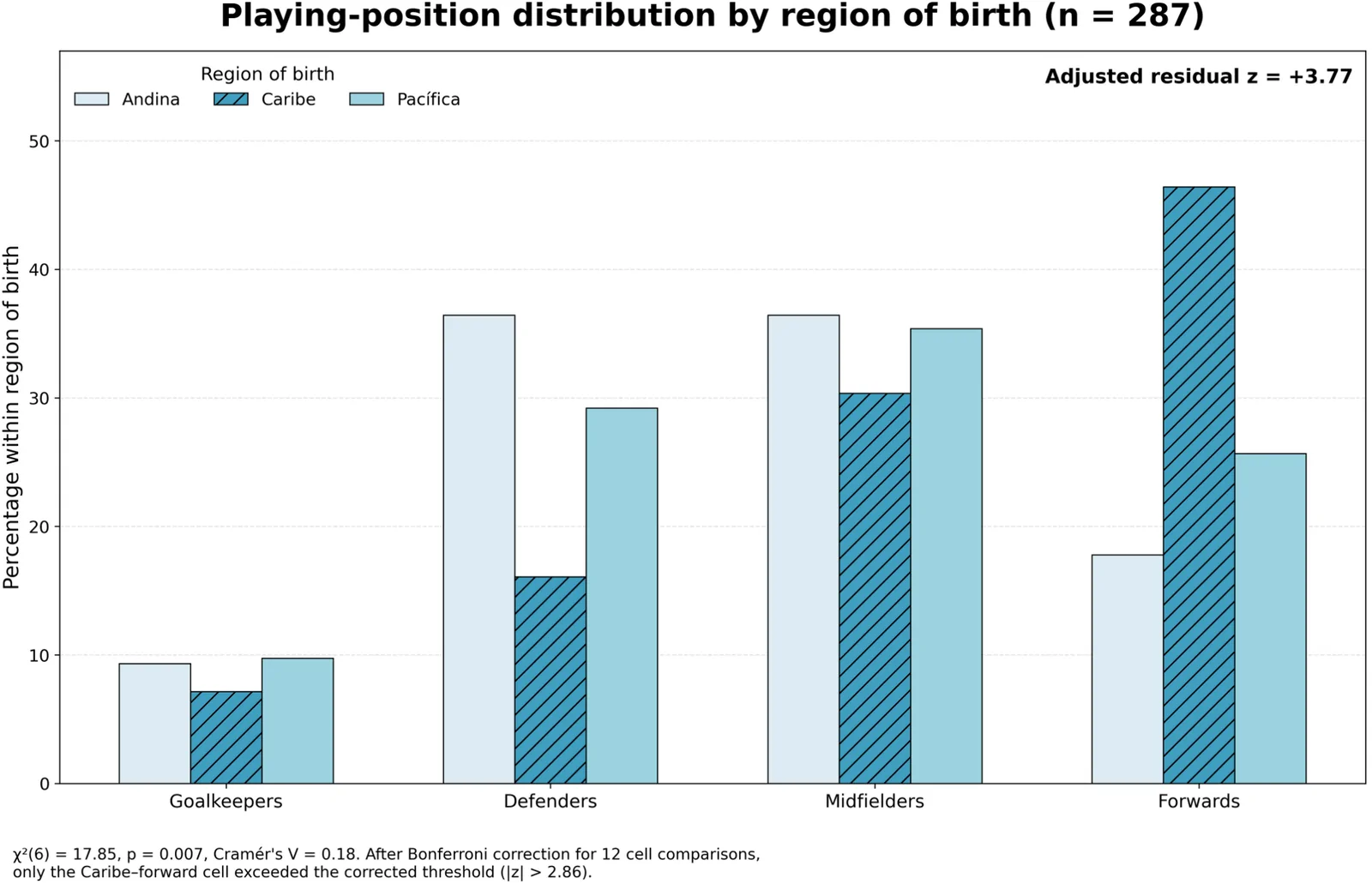
Playing-position distribution by region of birth, expressed as the percentage of players from each region in each of four squad-position categories (*n* = 287). After Bonferroni correction for twelve cell comparisons, only the Caribe–forward cell exceeded the corrected threshold (adjusted standardised residual *z* = 3.77; |z| > 2.86). The overall association was *χ*²(6) = 17.85, *p* = 0.007, Cramér's V = 0.18.

## 4 Discussion

Through the relational opportunity-structure framework, this study's central finding is a territorial mismatch: Colombian men's internationals originated across a broader departmental geography than that through which their first professional appearances were recorded. In the directly comparable domestic-debut sample, birthplaces covered 20 departments and debuts 14, while the three leading departments accounted for 58.4% and 70.1% of the two distributions, respectively; only 44.7% of players with verified paired data debuted in their department of birth. This concentration is institutionally meaningful, but it does not establish unequal selection probabilities. Two secondary findings qualify common narratives: regional composition fluctuated rather than moving progressively towards the Pacific and Caribbean regions, and the region-position association was small, with only the Caribe-forward cell surviving correction for multiple comparisons.

### 4.1 Dispersed origins and concentrated professional entry

The birthplace-debut contrast identifies two distinct geographies within the same national-team pool. Birthplace records the territorial distribution of origin, whereas professional debut marks the location at which entry into paid senior competition became observable. The narrower debut distribution is consistent with a labour market in which clubs, contracts and first-team opportunities are spatially concentrated, a pattern also reported in research on Brazilian elite futsal (10). The Colombian evidence does not reveal the full route between those markers, but it demonstrates that a birthplace map cannot stand in for the geography of professional access.

Professional debut is institutionally constituted rather than a neutral biographical event: clubs decide whom to register and field, and league geography determines where those decisions can occur. The concentration is therefore a property of institutional geography as well as individual pathways. It may reflect club location, academy capacity, scouting reach, travel and relocation support or selection thresholds. Because those processes were not measured, the result identifies a potential spatial bottleneck through which successful players passed; it does not demonstrate that access probabilities were unequal or that clubs reproduced territorial inequality.

The distinction is substantive as well as methodological. Academy research has shown that birthplace and place of sporting development frequently diverge and may relate differently to later progression (8). In the present data, a player could have grown up, trained or been recruited outside both his birth department and the department of senior debut. Consequently, the defensible claim is not that the debuting clubs produced every player they fielded, but that professional entry was recorded through fewer territories than geographical origin. This finding motivates direct measurement of upbringing, youth-club participation, academy location and migration timing rather than treating birthplace as a proxy for all four.

The descriptive departmental rates reinforce the need to separate origin from formal infrastructure. Chocó recorded the highest number of internationals per million inhabitants despite persistent multidimensional poverty and the absence of a department-based club among the professional Category A or B affiliates at the study cut-off (21, 22). This does not show that poverty or institutional scarcity generated elite players. It shows that high per-capita representation can coexist with limited local professional entry, while informal play, family support, community clubs and outward recruitment remain plausible but unmeasured contributors. More generally, formal clubs are only one component of a multidimensional development geography (23).

More broadly, this framework follows a relational sociological view in which opportunities arise from positions and transactions within structured fields rather than from isolated personal attributes (24); unequal access to economic, social and cultural capital can condition entry to valued positions and, when sustained institutionally, contribute to social reproduction (25). The present study identifies a spatial configuration in which such processes may operate but does not demonstrate their reproduction.

### 4.2 Temporal fluctuation rather than progressive regional replacement

The temporal analysis contradicts a simple narrative of progressive replacement of Andean origins by Pacific and Caribbean origins. Pacífica reached its highest share in the middle period and then fell below its initial proportion; Caribe fell in the middle period and later returned to approximately its starting share; Andina remained comparatively stable. Moving the second boundary from 2010 to Colombia's 2014 World Cup return changed the denominators but not the qualitative pattern. The result is therefore better described as historical fluctuation and recomposition than as a continuous directional transition.

This finding should not be overextended into an annual trend or a causal account of regional development. The bins contain different numbers of first selections and combine distinct tournament cycles and generations. Their value is to test the direction of a widely repeated historical proposition at a scale less volatile than individual tournaments. On that limited question, the evidence is informative: neither the original periods nor the 2014 sensitivity boundary supports monotonic growth in Pacific and Caribbean representation. Future work with annualised cohorts and time-varying population denominators could examine shorter-term changes more precisely.

### 4.3 Region and playing position as a secondary analysis

The positional analysis provides limited evidence rather than a regional profile. The global association was statistically significant but small, and only the excess of Caribe-born forwards remained after correction for twelve cell comparisons. This isolated pattern is compatible with positional-stacking research, which interprets uneven representation in relation to social classification, institutional expectations and recruitment or coaching practices rather than innate group differences (11–13). It does not validate broader stereotypes concerning Caribbean attacking identity, Pacific physicality or Andean tactical organisation.

Because the dataset contains no measures of coaches' beliefs, selection decisions, self-identified ethnicity, skin-colour classifications or detailed tactical roles, it cannot determine whether the Caribe–forward cell arose from coaching expectations, player self-selection, historical club structures, migration, tactical systems or chance variation that survived the stated correction. Regional stereotypes may nevertheless be institutionally consequential if they shape trial opportunities, positional assignment or specialised training and later distributions are treated as confirmation. This self-reinforcing mechanism is compatible with stacking theory but was not tested here. Region of birth should therefore not be used as evidence of expected aptitude or positional suitability. The result is retained as a transparent secondary analysis because it directly evaluates a recurrent discourse, but its interpretive weight remains deliberately limited.

### 4.4 Implications, limitations and future research

For federations, leagues, clubs and academies, birthplace and professional-debut maps answer different questions. The first indicates the territorial reach of elite origins; the second identifies where access to senior professional competition was first recorded. Comparing them can guide audits of scouting reach, trial opportunities, academy pathways and mobility support, especially for territories that contribute player origins but rarely host professional debuts. These retrospective counts do not demonstrate institutional bias or justify redistributing resources on their own, but they identify where pathway data should be collected and where institutional concentration warrants examination.

These recommendations primarily concern pathway governance rather than sport marketing. However, decisions concerning scouting, regional investment and talent pathways are made within a broader stakeholder environment encompassing organisational communication, sponsorship and corporate social responsibility (26, 27). Future research could examine whether communication and stakeholder-engagement practices amplify, reproduce or counter territorial narratives about football talent. The present study did not examine these processes.

Professional debut marks entry into paid competition, but it is not itself evidence of upward social mobility. Demonstrating such mobility would require information on family social origin, earnings, occupational security, career duration and intergenerational change, none of which was measured. The findings therefore neither confirm nor dismiss football's mobility potential. They show that successful internationals reached professional status through a narrower territorial geography than their birthplaces, qualifying any unexamined claim that opportunity is territorially open without establishing that football reproduced existing regional inequalities.

The next research step is longitudinal pathway reconstruction. Individual records should distinguish place of upbringing, municipality of sporting development, youth clubs, academy enrolment, age and distance at migration, trials, contract offers and the move immediately preceding professional debut (8, 9). Spatial models should then combine cohort-relevant denominators with population density, transport, safety, facilities, informal-play space and club proximity (4, 7). Such designs could test whether the observed concentration reflects scouting reach, mobility costs, academy capacity, selection thresholds or other mechanisms instead of inferring them from aggregate maps.

Several limitations bound the conclusions. Birthplace is not place of upbringing or development, and debut department is not a complete measure of training. The sample is conditioned on a highly selective outcome, selection for a senior major-tournament squad and contains no denominator of aspirants, unsuccessful trials or comparable performance by territory. It therefore cannot estimate territorial probabilities of professional entry, establish unequal access or measure upward social mobility. The design is observational and ecological; causal mechanisms and individual ethnic identity were not measured. Positions were reduced to four broad, historically variable categories. Twenty-one municipality-of-birth discrepancies remained unresolved, although departmental classification was unaffected, and missingness varied by outcome. Regional units are internally heterogeneous, population density was unavailable and the 1985 census denominator is a common descriptive benchmark rather than a cohort-standardised incidence denominator. The temporal periods are broad and unequal, although the 2014 sensitivity analysis reduces concern that the interpretation depends on a single boundary. Finally, the findings apply to Colombian men selected for senior major-tournament squads and should not be extended automatically to women's football, youth academies, domestic professionals or other sports.

## 5 Conclusion

Across twenty-seven major tournaments between 1962 and 2026, Colombian men's internationals originated across a broader departmental geography than that through which they entered professional football. In directly comparable domestic-debut cases, birthplaces spanned 20 departments and debuts 14, while only 44.7% of all players with verified paired data debuted in their birth department. Regional composition fluctuated across periods rather than shifting monotonically towards Pacific and Caribbean origins, including under an alternative 2014 boundary. The small positional association and single corrected Caribe–forward cell provide insufficient support for broader regional profiling. Through a relational opportunity-structure framework, the study identifies the institutional geography through which successful players entered paid competition; it does not by itself demonstrate unequal selection or social reproduction. Longitudinal records of upbringing, youth clubs, academies, migration, trials, unsuccessful applicants and socioeconomic outcomes are required to determine whether concentrated professional gateways translate into unequal territorial opportunity or constrain football's potential as a route of social mobility.

## Data Availability

The coded dataset, source and decision log, departmental-rate data, historical-period summaries and reproducible Python code are openly available in Zenodo at https://doi.org/10.5281/zenodo.22070374. The underlying documentary records are publicly accessible through the source URLs recorded in Supplementary Data S1; no restricted data were used. Copies of the study files are also provided as Supplementary Material.

## Supplementary material

Supplementary Data S1

Coded dataset, source log, coding documentation and decision log.

Supplementary Data S2

Departmental population denominators and descriptive rates.

Supplementary Data S3

Primary historical-period counts and percentages, alternative 2014-boundary sensitivity summary and method notes.

Supplementary File S4

Reproducible Python code, including paired territorial concentration, region-by-position analyses and both temporal summaries.
